# The Proteome of Circulating Large Extracellular Vesicles in Diabetes and Hypertension

**DOI:** 10.3390/ijms24054930

**Published:** 2023-03-03

**Authors:** Akram Abolbaghaei, Maddison Turner, Jean-François Thibodeau, Chet E. Holterman, Christopher R. J. Kennedy, Dylan Burger

**Affiliations:** 1 Chronic Disease Program, Kidney Research Centre, Ottawa Hospital Research Institute, Ottawa, ON K1H 8M5, Canada; 2 Departments of Medicine and Cellular and Molecular Medicine, University of Ottawa, Ottawa, ON K1H 8M5, Canada; 3 School of Pharmaceutical Sciences, University of Ottawa, Ottawa, ON K1H 8M5, Canada

**Keywords:** diabetes, hypertension, vascular, extracellular vesicles, microparticles, microvesicles, cardiovascular

## Abstract

Hypertension and diabetes induce vascular injury through processes that are not fully understood. Changes in extracellular vesicle (EV) composition could provide novel insights. Here, we examined the protein composition of circulating EVs from hypertensive, diabetic and healthy mice. EVs were isolated from transgenic mice overexpressing human renin in the liver (TtRhRen, hypertensive), OVE26 type 1 diabetic mice and wild-type (WT) mice. Protein content was analyzed using liquid chromatography–mass spectrometry. We identified 544 independent proteins, of which 408 were found in all groups, 34 were exclusive to WT, 16 were exclusive to OVE26 and 5 were exclusive to TTRhRen mice. Amongst the differentially expressed proteins, haptoglobin (HPT) was upregulated and ankyrin-1 (ANK1) was downregulated in OVE26 and TtRhRen mice compared with WT controls. Conversely, TSP4 and Co3A1 were upregulated and SAA4 was downregulated exclusively in diabetic mice; and PPN was upregulated and SPTB1 and SPTA1 were downregulated in hypertensive mice, compared to WT mice. Ingenuity pathway analysis identified enrichment in proteins associated with SNARE signaling, the complement system and NAD homeostasis in EVs from diabetic mice. Conversely, in EVs from hypertensive mice, there was enrichment in semaphroin and Rho signaling. Further analysis of these changes may improve understanding of vascular injury in hypertension and diabetes.

## 1. Introduction

Diabetes and hypertension are leading causes of cardiovascular disease (CVD) [1,2,3,4]. The two conditions may present independently or concomitantly, in which case, they synergistically increase cardiovascular risk. In this regard, the prevalence of hypertension is two-times higher in individuals with diabetes compared with those without diabetes [5], and the cardiovascular risk with diabetes is exacerbated by coexistent hypertension [5]. Substantial overlap in etiology and disease mechanisms has been reported between the two conditions, including the involvement of oxidative stress, the renin–angiotensin–aldosterone system, sympathetic nervous system dysregulation, adipokines and peroxisome proliferator-activated receptor signaling [6]. Nevertheless, there are also distinct pathways that are unique to diabetes or hypertension that may also cause vascular injury. Management of cardiovascular risk in these distinct but overlapping conditions requires a clear understanding of the molecular pathogenesis. However, despite significant progress in the understanding of the pathophysiology, the molecular alterations that mediate the initiation and progression of cardiovascular disease in diabetes and hypertension are not fully understood.

Circulating large extracellular vesicles (L-EVs) are novel biomarkers of cellular stress/injury [7,8]. L-EVs are 0.1–1.0 μm vesicles shed from the surfaces of cell membranes under conditions of stress [9,10]. Once formed, L-EVs contain membrane and cytosolic protein, mRNA and miRNA typical of their cell of origin, but lack nuclear material. They also play a crucial role in cell-to-cell communication, as they may physically interact and transfer lipids, proteins and nucleic acids from a cell of origin to recipient cells [9]. Crucially, they are present in biological fluids such as urine, blood, saliva and breast milk, and reflect a molecular fingerprint of the releasing cell type [9,11]. The protein composition of some circulating L-EVs may therefore provide more insight into the molecular changes in their cell of origin than analysis of whole plasma. This, in turn, could identify key molecular changes that contribute to vascular injury in diabetes and hypertension. While all types of EVs may provide insight into the cell of origin, L-EVs are particularly suitable for identification of altered pathways in disease, since the majority arise directly from stressed/injured cells [9,12].

The unbiased assessment of protein changes in diabetes has been employed in an effort to identify the dysregulated signaling responsible for diabetic complications. Numerous proteomic studies on diabetic plasma have been conducted, and candidate proteins such as TNFAIP6, CDNF, WIF1 and TGFbR3 have been described as possibly involved in the pathogenesis of vascular injury in diabetes [13,14,15]. Plasma protein profiling of newly diagnosed type 2 diabetes revealed proteins altered at the very early stage, reflecting key metabolic syndrome features, such as insulin resistance, adiposity, fatty liver disease and hyperglycemia [15]. Similarly, the plasma proteome of patients with type 1 diabetes with diabetic nephropathy revealed new putative biomarkers of kidney injury, such as transthyretin, apolipoprotein A1, apolipoprotein C1 and cystatin C [16]. Another study observed that type 1 diabetes was associated with the upregulation of six proteins (prothrombin, alpha-2-macroglobulin, apolipoprotein A-II, β2 glycoprotein I, Ig alpha-2 chain C region and alpha-1-microglobulin) and the downregulation of two proteins (complement C4 and pregnancy zone protein) [17]. In contrast, the number of studies that define proteomic signatures of hypertension is comparatively small. A recent study employed proteomics on plasma from individuals that were hypertensive and matched healthy controls [18]. The study identified 27 molecular alterations, such as osteocalcin, nexilin and phosphoinositide 3-kinase regulator 1; and pathway alterations, including atherogenesis, cellular calcium metabolism, cytoskeletal organization and angiogenesis [18,19,20]. Similarly, a plasma proteomics classifier based on a series of protein changes has been shown to improve risk prediction associated with renal disease in individuals with type 2 diabetes and hypertension [21].

Recently, several groups have examined the proteome of circulating EVs as a strategy to more specifically identify molecular alterations from stressed cells. For example, L-EVs from the plasma of individuals diagnosed with type 2 diabetes are enriched in proteins involved in cell adhesion, inflammation and platelet activation, such as S100A8, S100A9 and CD41 [22]. Interestingly, assessment of circulating EVs in plasma samples from women with gestational diabetes mellitus (GDM) showed altered protein expression as compared to healthy control through a shift towards proteins involved in metabolism, energy production and inflammation [23]. These studies suggest that there is alteration of the EV proteome in diabetes. However, further study and validation of differentially expressed proteins is necessary. Moreover, the EV proteome has not been examined in the context of hypertension. Thus, the aim of this study was to examine the effects of hypertension and diabetes on the molecular composition of circulating EVs, focusing on the L-EV subpopulation.

## 2. Results

### 2.1. Physiological and Biochemical Measures

Physiological parameters of healthy (*n* = 3), OVE26 (diabetic, *n* = 3) and TTRhRen (hypertensive, *n* = 3) mice, including blood pressure, blood glucose, heart weight, urinary albumin/creatinine and body weight, are presented in Table 1. As expected, blood pressure was elevated in TTRhRen mice, and blood glucose was higher in OVE26 mice, which is consistent with the expected phenotype of these two models. The urinary albumin/creatinine ratio was increased in OVE26 mice. A reduction was also observed in body weight in OVE26.

### 2.2. Characterization of EV Isolates

**Figure 1:**
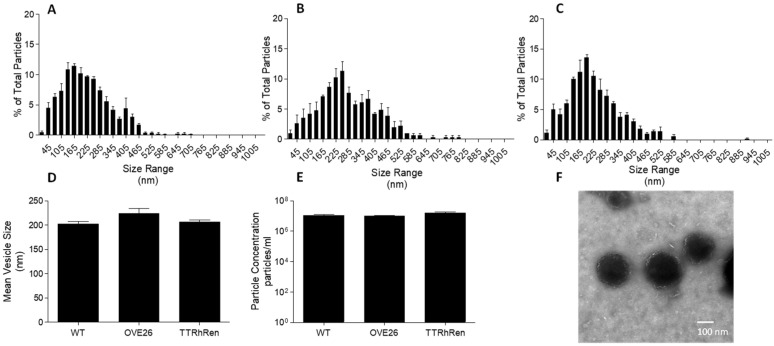
Nanoparticle tracking analysis and transmission electron microscopy of large EV isolates. Shown are size distributions of L-EVs from WT (**A**), OVE26 (**B**), and TTRhRen (**C**) mouse plasma. (**D**) Comparison of mean particle size by NTA (P = NS, *n* = 3). (**E**) Comparison of particle concentration by NTA (P = NS, *n* = 3). (**F**) Representative transmission electron micrograph of pooled plasma showing distinct vesicle size and shape.

Following differential centrifugation, EV isolates were assessed for size and morphology. Nanoparticle tracking analysis revealed a population of EVs with minimal presence of vesicles less than 100 nm in size (Figure 1A–D). We did not observe differences in EV size or concentration among treatment groups (Figure 1D,E). Transmission electron microscopy analysis showed vesicles approximately 150 nm in size with intact membranes (Figure 1F). Western blot analysis confirmed the presence of vesicle markers flotillin-1 and TSG-101 (Supplementary Figure S1).

### 2.3. Proteomics Analysis and Associated Signaling Pathways

**Figure 2:**
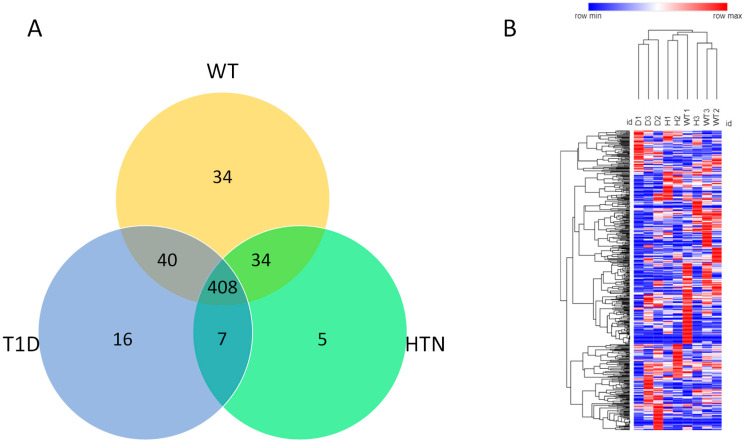
Venn diagram represents the differences in protein composition among diabetes, hypertension, and wild-type mice (**A**). Protein profile analysis of differentially expressed proteins (Hierarchical clustering) across all three groups of mice using Morpheus (https://software.boardinstitute.org/morpheus/, (accessed on 1 June 2022)) (**B**).

To gain insight into the molecular changes associated with hypertension and diabetes, we next examined the protein composition of isolated circulating EVs. Among all samples, LC–MS/MS analysis identified 544 proteins with a minimum of two spectral counts per sample with a 95% peptide threshold and a 99% protein threshold. Of the 544 proteins identified, 408 were common to all groups, whereas 34 were exclusive to healthy mice, 5 to hypertensive mice and 16 to diabetic mice. Seven proteins were common in diabetes and hypertension groups, 34 were common to healthy and hypertension groups and 40 were common to healthy and diabetic mice (Figure 2A).

Notably, in hierarchical clustered heatmaps, we observed separation according to disease, confirming that molecular profiles of EVs are most similar within disease conditions and suggesting that EVs may reveal disease-specific protein alterations (Figure 2B).

**Figure 3:**
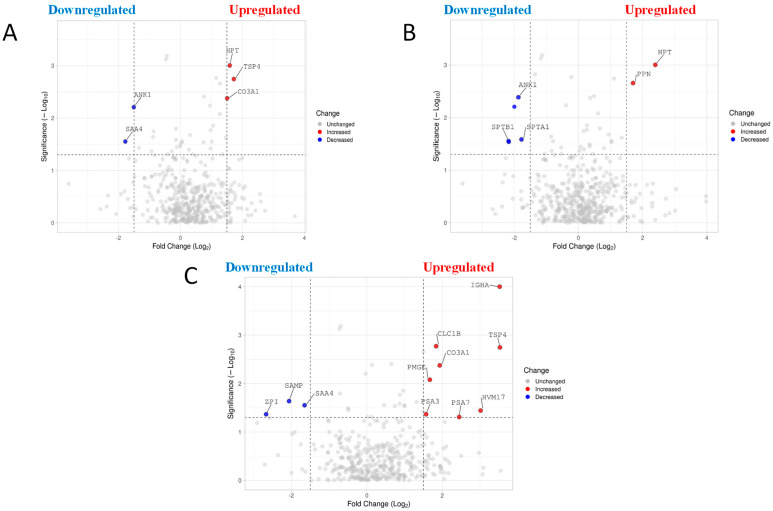
Volcano plot representing differentially expressed protein comparisons. (**A**) Differentially expressed protein in diabetes plasma L-EVs compared to wild-type plasma L-EVs. The horizontal axis represents the log2 of fold change and the vertical axis represent *p*-value. Each gray dot represents a protein with red dots on the right above the dashed line are proteins upregulated while the blue dots on the left are downregulated. (−log *p* value of 1.3010299957 is considered significant as it translates to a *p* value of 0.05). VolcaNoseR. https://goedhart.shinyapps.io/VolcaNoseR/, (accessed on 15 April 2022). (**B**) Volcano plot representing differentially expressed protein in hypertensive plasma L-EVs compared to wild-type L-EVs. (**C**) Volcano plot representing differentially expressed protein in diabetes plasma L-EVs compared to hypertensive plasma L-EVs.

The relative abundance of L-EV protein in diabetic mice in comparison to healthy mice is presented as a volcano plot in Figure 3A. A total of five differentially expressed proteins were identified (Table 2). Of these proteins, three were upregulated (TSP4, HPT, CO3A1) and two were downregulated (ANK1, SAA4) (Figure 3A, Table 2).

With respect to hypertensive mice in comparison to healthy, a total of five differentially expressed proteins were identified (Table 3). Of these proteins, two proteins were upregulated (HPT, PPN) and three were downregulated (ANK1, SPTB1, SPTA1) (Figure 3B, Table 3).

Finally, for diabetic mice in comparison to hypertensive mice, a total of 11 differentially expressed proteins were identified in EVs (Table 4). Eight proteins were upregulated (IGHA, TSP4, CLC1B, HVM17, CO3A1, PSA3, PSA7,PMGE), and three proteins were downregulated (ZPI, SAMP, SAA4) (Figure 3C, Table 4).

### 2.4. Protein Ingenuity Pathway Analysis

To further understand the impacts of diabetes and hypertension on the circulating L-EV proteome, Ingenuity Pathway Analysis (IPA) software was used to assess “diseases and functions”, and “canonical pathways” of all identified proteins.

**Figure 4:**
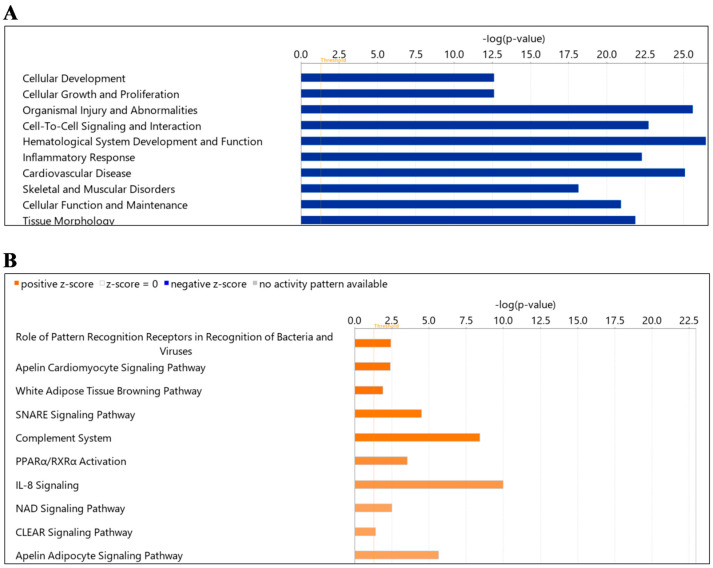
Summary of Ingenuity Pathway Analysis (IPA) for L-EV proteins in diabetes as compared to wild-type. Shown are disease and function (**A**) and canonical pathways (**B**). The dotted orange line represents the threshold of significance (*p* = 0.05).

Using all proteins in EVs from diabetes compared to healthy mice, IPA noted enrichment in “diseases and function” for cellular development, cellular growth and proliferation, organismal injury and abnormalities, cell-to-cell signaling and interaction, hematological system development and function, inflammatory response, cardiovascular diseases, skeletal and muscular disorders, cellular function and maintenance and tissue morphology (Figure 4A). Similarly, IPA for “canonical pathways” identified pattern recognition, apelin cardiomyocyte signaling, white adipose tissue browning, SNARE signaling, complement system, PPARα, RxR α activation, IL-8 signaling, NAD homeostasis and CLEAR signaling as enriched in diabetes (Table 5). Among these pathways, the apelin cardiomyocyte signalling pathway, white adipose tissue browning pathway, apelin adipocyte signaling pathway, PPARα, RxR α activation and the IL-8 signaling pathway were enriched pathways associated with inflammation in this group. Other pathways, such as the SNARE signaling pathway, are involved in extracellular vesicle formation or mediate vesicle fusion. The NAD signaling pathway is involved in mitochondrial biogenesis, and the CLEAR signaling pathway is responsible for lysosomal activity (lysosomal expression and regulation) (Figure 4B; Table 5). As shown in Table 5, significantly changed proteins participating in these pathways included complement-related ones (C1QA/C1QB), myosin (MYH10, MYH14, MYH9), mitochondrial proteins (ACADL, ACADM), etc.

**Figure 5:**
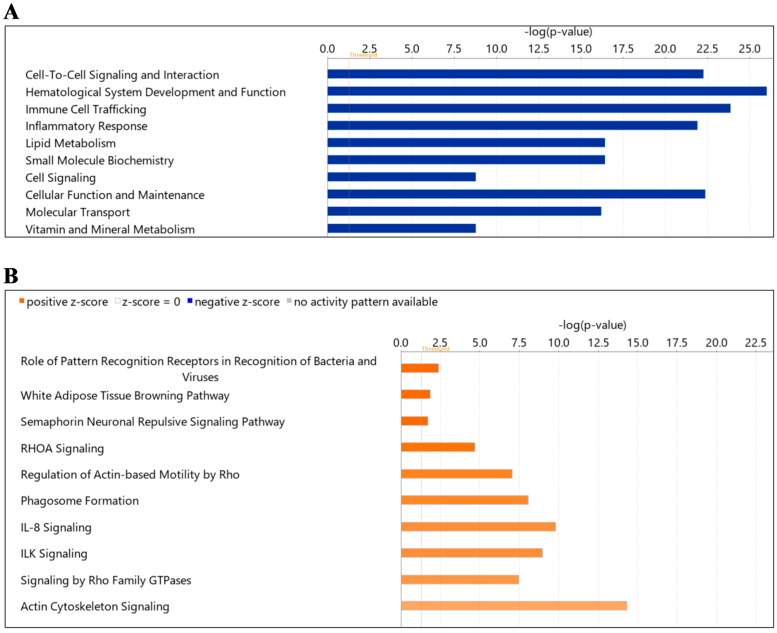
Summary of Ingenuity Pathway Analysis (IPA) for L-EV proteins in hypertension as compared to wild-type. Shown are disease and function (**A**) and canonical pathways (**B**). The dotted orange line represents the threshold of significance (*p* = 0.05).

Next, we examined the “diseases and function” in hypertension compared to healthy. Cell-to-cell signaling and interaction, hematological system development and function, immune cell trafficking, inflammatory response, lipid metabolism, small molecule biochemistry, cell signaling, cellular function and maintenance, molecular transport, vitamin and mineral metabolism were noted as significantly enriched (Figure 5A). For “canonical pathways” the top pathways included signaling pathways such as pattern recognition receptor, white adipose tissue browning, semaphorin neuronal repulsive, RhoA signaling, regulation of Actin-based motility by Rho, phagosome formation, IL-8 signaling, ILK signaling, signaling by Rho family GTPases and actin cytoskeleton (Figure 5B, Table 6). The “canonical pathways” included as white adipose tissue browning pathway (CAMP, LDHA, LDHB, THRB) and IL-8 signaling pathway (CDC42, EGFR, GNA13, GNAI2, GNQ, GNAZ, MMP2, MYL9, RAC1, RAC2, RAP1A, RAP1B, RHOA, VCAM1).

## 3. Discussion

Vascular injury and endothelial dysfunction are common features of both hypertension and diabetes. However, as pathogenic mechanisms driving such changes may differ between the two conditions, the approaches to therapeutic management of vascular injury may also differ. The present study examined the effects of hypertension and diabetes on the molecular composition of circulating L-EVs as an indirect measure of vascular alterations. Using well-established mouse models, we observed distinct protein signatures in EV populations and the greatest agreement within disease conditions. Further assessment with IPA identified enrichment in key signaling pathways, including apelin and SNARE signaling (diabetes) and semaphorin and Rho signaling (hypertension). Our results suggest that EV protein composition is reflective of the underlying molecular changes driving disease pathogenesis.

In this study, we observed common/distinct changes in proteins in diabetic vs. healthy mice. This study identified a total of five differentially expressed proteins in diabetic mice compared with healthy mice. Of these proteins, three were upregulated (TSP4, HPT, CO3A1) and two were downregulated (ANK1, SAA4). Some of these changes have been identified in other studies and are in accordance with our observations [24,25,26,27,28]. Thrombospondin-4 (TSP4) has been shown previously to cause peripheral arterial disease in diabetes [24]. The fact that TSP4 was elevated in our vesicles, suggests activation of a pathway that may contribute to this process. Increased amounts of type-III collagen (CO3A1) have been noted in tubular epithelial cells in individuals with diabetic nephropathy; however, to the best of our knowledge, this has not been reported in the vasculature [25]. While previous reports have shown elevation in haptoglobin (HPT) in individuals with elevated glucose and metabolic syndrome [26], alterations in ANK-1 do not appear to have been reported previously [27]. Based on the protein composition of EVs, IPA identified the canonical pathways that are most enriched in diabetic vs. control mice. These pathways included apelin signaling, white adipose tissue browning, SNARE signaling, complement activation, PPARα and NAD biogenesis. Previous studies have reported that apelin (a peptide hormone linked with obesity and diabetes) and its receptor inhibit vascular injury in diabetes, including the endocrine response to stress, lipid metabolism, homeostasis and angiogenesis [28,29]. It is possible that enrichment in apelin signaling is a protective mechanism to limit vascular injury in diabetic mice. SNARE proteins are involved in insulin granule exocytosis, but less is known about their relevance to vascular health [30,31,32,33,34] in diabetes. However, SNARE complexes also facilitate EV release, and it is possible that their enrichment is simply a result of altered EV release under diabetic conditions [35]. PPARα signaling has been shown to lower blood pressure and reduce oxidative stress [36,37,38]. The enrichment in this signaling may therefore be evidence of a protective response. Finally, enrichment in proteins related to NAD+ biogenic pathways may be evidence of dysregulation of this pathway, as has been reported in animal and human diabetes [39].

We also examined changes in proteins in EVs between hypertensive mice and healthy ones. A total of five differentially expressed proteins were also identified in this group: two proteins were upregulated (PPN, HPT), and three proteins were downregulated (ANK1, SPTB1, SPTA1). Interestingly, the upregulation of HPT and downregulation of ANK1 were also seen in our diabetes mice, suggesting that these may be common pathways involved in vascular injury in both conditions. Conversely, the upregulation of PPN and downregulation of SPTB1 and SPTA1 were unique to hypertension. In addition to the previously described relationship with blood glucose, increases in HPT have been shown in individuals with elevated blood pressure and metabolic syndrome [26]. Mechanistically, HPT has been shown to lower blood pressure in a model of hemoglobin-induced hypertension [40]. Thus, increased HPT may be a common protective pathway activated in both hypertension and diabetes. When examining the protein composition of EVs from hypertensive and healthy mice, IPA identified canonical pathways that are most enriched in hypertension. These pathways included semaphorin neuronal repulsive signaling, RhoA signaling, phagosome formation, ILK signaling and actin cytoskeleton signaling. RhoA/Rho kinase signaling has long been implicated in hypertension due to its important role in smooth muscle contraction [41,42,43]. Thus, it is perhaps not surprising that this pathway was elevated in EVs from hypertensive mice in our study. As RhoA/Rho kinase also plays important roles in cytoskeletal regulation [44] and phagosome formation [45], enrichment in these pathways may be related to convergent signaling. Interestingly, integrin-linked kinase (ILK) signaling has been implicated in hypertension-mediated organ damage [46,47]. However, to the best of our knowledge, semaphorin signaling has not been implicated in blood pressure regulation and may represent a novel pathway for future study.

Our study identified over 500 proteins in circulating L-EVs. The vast majority of those proteins (>400) were found in all groups. Interestingly, only a small number of proteins were found to be exclusive to a particular disease state. These proteins could represent those which were altered in response to the disease condition, or they may have actively contributed to disease pathogenesis. Future research should seek to clarify the roles of these proteins as biomarkers or pathogenic mediators of hypertensive or diabetic vascular injury. Interestingly, our hierarchical clustering algorithm largely separated our L-EV isolates based on disease state. L-EVs from diabetic mice were distinctly categorized, and those from hypertension and wild-type mice were more closely overlapping in protein signatures. While there were large variations within each group, it is reassuring that the greatest similarities were seen within the same experimental group. Whether this will remain the case with larger and more heterogeneous populations (i.e., human cohorts) remains to be seen.

The present work represents one of the earliest to examine distinct proteomic changes in diabetic and hypertensive mice and the first to employ EVs as a tool to facilitate this analysis. One of the strengths of this study is the use of well-defined mouse models of diabetes and hypertension. In addition, the inclusion of both hypertension and diabetic mice allowed for the identification of both common and unique enriched pathways that may be contributing to disease pathogenesis and progression. We focused our efforts on the L-EV subpopulation due to the tight linkage between their formation and cellular stress [48]. Nevertheless, there is abundant evidence that other EV populations such as small EVs/exosomes play a role in cardiovascular physiology [49,50]. Future studies should strive to clarify the impacts of diabetes and hypertension on other EV populations. Our study also had some limitations to consider. First, relatively few male mice were studied, and although we did observe greatest similarity within disease, it is likely that the degree of heterogeneity was underestimated. Second, our observations require validation, and the potential for therapeutic targeting of dysregulated pathways is not known at this time. It is also worth noting that the approach to assessing protein signatures in circulating EVs does not provide a complete picture of molecular changes such as epigenetic alterations. Finally, there is also potential for differences in hypertension and diabetes-associated changes between mice and humans. Thus, independent validation in humans is a logical next step. Nevertheless, our results suggest that circulating EVs may be used to assess protein changes to the vasculature in a minimally invasive fashion.

## 4. Materials and Methods

### 4.1. Animals

Mouse models of hypertension, type 1 diabetes and their wild-type (WT) littermates (healthy control) were employed on an FVB/N background, and male mice were studied at 20 weeks of age. Hypertensive TTRhRen mice express a modified human pro-renin transgene under the control of the mouse transthyretin promoter [51,52,53]. These mice overexpress human renin, and hemizygotes exhibit elevated systolic blood pressure and cardiac hypertrophy by 4 months of age. To model type 1 diabetes, we employed the transgenic OVE26 mice which have a pancreatic beta cell-specific overexpression of a calmodulin mini-gene and are insulinemic from birth [54]. Hypertensive and diabetic mice, and their healthy littermates, were housed at the University of Ottawa Animal Care Facility with free access to food and water. Protocols were approved by the University of Ottawa Animal Care Committee and conducted in accordance with the guidelines of the Canadian Council on Animal Care.

### 4.2. Blood Pressure Measurement

Blood pressure was assessed by tail cuff plethysmography (BP 2000, Visitech Systems, Apex, NC, USA), as described previously [51,52,55]. Following a five-day training period (10 BP readings/day), weekly BP measurements were obtained beginning at 10 weeks.

### 4.3. Physiological Parameters

Immediately prior to sacrifice, spot urine samples were collected and centrifuged at 2500× *g* for 10 min and stored at −80 °C. Urinary albumin was assessed with the Mouse Albumin Elisa Kit (Bethyl Labs, Montgomery, TX, USA), following the manufacturer’s protocol. Albumin levels were normalized to creatinine concentration using the Creatinine Companion Kit (Exocell, Philadelphia, PA, USA).

At sacrifice, blood samples were collected into heparinized syringes by cardiac puncture and immediately centrifuged at 2500× *g* for 10 min at 4 °C. Plasma glucose levels were determined by glucometry (Bayer Contour), and remaining plasma was used for EV isolation. Tibias, kidneys, and hearts were removed and weighed. Organ weights were normalized to tibia length.

### 4.4. EV Isolation

Circulating L-EVs were isolated via differential centrifugation from plasma by centrifugation for 20 min at 20,000× *g* to obtain a L-EV-rich pellet. The isolated vesicles were washed with 1× PBS and re-suspended in PBS (nanoparticle tracking analysis), 2.5% glutaraldehyde in PBS (transmission electron microscopy), or RIPA buffer (proteomics) [10,56].

### 4.5. Nanoparticle Tracking Analysis

To confirm the presence of vesicles between 100 and 1000 nm in diameter (L-EVs) in the vesicle isolates, nanoparticle tracking analysis (NTA) was conducted to assess vesicle size. Briefly, samples were diluted in 1× PBS to the working range of the system and analyzed on a ZetaView PMX110 (Particle Metrix, Meerbusch, Germany) in size mode, as we have done previously [57,58,59,60].

### 4.6. Electron Microscopy

EVs were examined by transmission electron microscopy (TEM), as described previously [59,61]. In brief, L-EVs were isolated from pooled plasma samples and fixed with 2.5% glutaraldehyde in PBS for four hours at room temperature. Next, the pellet was washed in 0.1 M Na cacodylate buffer, post-fixed in 2% OsO4 and dehydrated in a series of graded ethanol dilutions. Samples were embedded in Spurr Resin, and 60 nm sections were prepared on copper grids. Samples were visualized using a JEOL JEM-1400 Plus electron microscope (JEOL Ltd, Tokyo, Japan).

### 4.7. Western Blot Analysis

L-EV isolates from pooled plasma samples were examined for the presence of vesicle protein markers by Western blot analysis, as described previously [59,62]. Protein lysates were 10% polyacrylamide gels and levels of the vesicle-associated proteins flotillin-1 (1:2000, BD Biosciences, Franklin Lakes, NJ, USA) and TSG101 (1:2000, Abcam Inc., Toronto, ON, Canada) were assessed.

### 4.8. Proteomic Assessment of EVs

EV isolates were separated by gel electrophoresis on a 4–15% Mini PROTEAN TGX Gel. Separated proteins were excised by a gel excision tool (The Gel Company, San Francisco, CA, USA) and placed in 1% acetic acid. In-gel proteins were digested with trypsin, purified by ZipTip, concentrated in an Eppendorf vacufuge (ThermoFisher Scientific, Nepean, ON, Canada) and re-suspended in 0.1% formic acid.

Digested peptides were then analyzed by label-free LC-MS/MS through the OHRI Proteomics Core Facility, as described previously [63]. Briefly, the system consisted of an UltiMate 3000 RSLC nano HPLC, LTQ Orbitrap XL hybrid mass spectrometer (ThermoFisher Scientific, Nepean, ON, Canada) the XCalibur software (version 2.0.7) and a nanospray ionization source. Peptides were eluted over a 60 min gradient of 3–45% acetonitrile at a flow rate of 300 nL/min through a 10 cm long column with integrated emitter tip (Picofrit PF360-75-15-N-5 from New Objective packed with Zorbax SB-C18, 5 micron from Agilent, Santa Clara, CA, USA). MS scans were acquired in FTMS mode at a resolution setting of 60,000. MS2 scans were acquired in ion-trap CID mode using data-dependent acquisition of the top 5 ions from each MS scan. MASCOT software (Matrix Science, Boston, MA, USA, version 2.5.1) was used to infer peptides and proteins from the observed MS/MS spectra and matched against mouse sequences from SwissProt. Mass tolerance parameters were MS ±10 ppm and MS/MS ±0.6 Da. Enzyme specificities were set to “Trypsin” with ≤2 miscuts; variable modifications was set to oxidation of methionine, protein N-terminal acetylation, pyrocarbamidomethlyation of N-terminal cysteine and conversion of glutamine to pyroglutamate; and fixed modifications was set to carbamidomethylation of cysteine. “Identified MASCOT peptides and proteins were confirmed using Scaffold (Proteome Software Inc., Portland, OR, USA version Scaffold_4.7.3, Proteome Software Inc., Portland, OR, USA)” 79. The scaffold FDR algorithm accepted peptides with a greater than 95% probability, and proteins were accepted if they contained at least 2 identified peptides and had a greater than 99% probability.

The differences in protein composition among diabetes, hypertension and healthy mice were identified using Functional Enrichment analysis tool (FunRich version 3.1.4), an open access, standalone functional enrichment and interaction network analysis tool and presented as a Venn diagram [64].

### 4.9. Bioinformatics Analysis

For hierarchical clustered heatmaps, Z-scores of log2 protein abundances (Normalized total spectra) were first calculated, and column clustering was calculated using the linkage function (metric = “Euclidean distance”, Linkage method = “average”) with column clustering through MORPHEUS by Broad Institute (RRID:SCR_017386), a software tool for versatile matrix visualization. (https://software.broadinstitute.org/morpheus, (accessed on 1 June 2022)).

A volcano plot of log2 fold change versus −log10 (significance) of differentially expressed proteins comparing diabetes, hypertension and healthy mice was made using VolcaNoseR (https://huygens.science.uva.nl/VolcaNoseR (accessed on 1 June 2022)) [65] with a −log *p* value (a –log *p* value of <1.3010299957, corresponding to *p* < 0.05 was considered significant) and the fold change threshold of 1.5.

Ingenuity Pathway Analysis (IPA) software (Ingenuity Systems, Mountain View, CA, USA; www.ingenuity.com, (accessed on 17 March 2022)) was used to identify “diseases and functions” and “canonical pathways” that are most significant to the dataset and to categorize differentially dysregulated proteins in specific diseases and functions for the proteins exclusive to three different types of mice. The pathways and diseases with *p* < 0.05 were listed and considered significantly different.

### 4.10. Statistical Analysis

To analyze differences in physiological parameters between hypertensive, diabetic and healthy mice, a one-way ANOVA was performed followed by Bonferroni correction test [66]. All statistical analyses were conducted using GraphPad Prism version 8.4.2 (GraphPad Software, La Jolla, CA, USA). Statistical significance was considered when *p* < 0.05.

## 5. Conclusions

In summary, circulating L-EVs have distinct molecular compositions that are dependent on pathogenic state. We also observed changes that were common to both hypertension and diabetes, and disease-specific changes. Further analysis of these changes may lead to the identification of novel pathways associated with the pathogenesis of vascular injury in hypertension and diabetes. Such knowledge is critical to optimizing and personalizing therapeutic management of vascular injury in these two conditions.

## Figures and Tables

**Table 1:** Physiological and biochemical measures.

Background	Blood Pressure (mmHg)	Blood Glucose (mg/dL)	Heart Weight/Tibia Length (mg/mm)	Urinary Albumin/Creatinine (ug/mg)	Body Weight (g)
**Wild-type**	114 ± 6	11.3 ± 0.7	8.4 ± 0.1	245 ± 69	32.4 ± 1.2
**OVE26**	124 ± 5	29.9 ± 0.8 *	7.3 ± 0.5	1026 ± 204 *	27.3 ± 0.9 *
**TTRhRen**	144 ± 8 *	12.3 ± 1.0	9.3 ± 0.6	504 ± 166	32.3 ± 1.1

* *p* < 0.05 vs. wild-type.

**Table 2:** Differentially expressed proteins in diabetes as compared to wild-type mice.

Protein	Change	Fold Change (log2)	*p* Value
**TSP4**	↑	1.72	0.0018
**HPT**	↑	1.59	0.00099
**CO3A1**	↑	1.51	0.0042
**ANK1**	↓	−1.51	0.0062
**SAA4**	↓	−1.78	0.028

↑ represents upregulated proteins. ↓ represents downregulated proteins. Proteins were selected using a cutoff point of *p* < 0.05.

**Table 3:** Differentially expressed proteins in hypertension as compared to wild-type mice.

Protein	Change	Fold Change (log2)	*p* Value
**HPT**	↑	2.40	0.00099
**PPN**	↑	1.70	0.0022
**ANK1**	↓	−1.87	0.0041
**SPTB1**	↓	−2.18	0.028
**SPTA1**	↓	−1.78	0.026

↑ represents upregulated proteins. ↓ represents downregulated proteins. Proteins were selected using a cutoff point of *p* < 0.05.

**Table 4:** Differentially expressed proteins in diabetes as compared to hypertensive mice.

Protein	Change	Fold Change (log2)	*p* Value
**IGHA**	↑	3.51	0.0003
**TSP4**	↑	3.53	0.0003
**CLC1B**	↑	1.84	0.0146
**HVM17**	↑	3.01	0.001
**CO3A1**	↑	1.93	0.0117
**PSA3**	↑	1.57	0.027
**PSA7**	↑	2.45	0.00359
**PMGE**	↑	1.67	0.02148
**SAMP**	↓	−2.06	0.11502
**SAA4**	↓	−1.65	0.02237
**ZPI**	↓	−2.67	0.00215

↑ represents upregulated proteins. ↓ represents downregulated proteins. Proteins were selected using a cutoff point of *p* < 0.05.

**Table 5:** Top 10 canonical pathways and related proteins in diabetes compared with wild-type mice.

Canonical Pathway	Log (*p* Value)	Ratio	Z-Score	Proteins
**Role of Pattern Recognition Receptors in Recognition of Bacteria and Viruses**	2.45	0.0321	2	C1QA, C1QB, C1QC, MBL2, TGFB1
**Apelin Cardiomyocyte Signaling Pathway**	2.40	0.0404	2	GNAI2, MYL6, MYL9, TGFB1
**White Adipose Tissue Browning Pathway**	1.90	0.029	2	CAMP, LDHA, LDHB, THRB
**SNARE Signaling Pathway**	4.51	0.0515	1.89	MYH10, MYH14, MYH9, MYL6, MYL9, RAB6A, RAB7A
**Complement System**	8.43	0.189	1.89	C1QA, C1QB, C1QC, C4BPA, MASP1, MASP2, MBL2
**PPARα/RXRα Activation**	3.53	0.0359	1.633	ACADL, APOA1, APOA2, GNAQ, RAP1A, RAP1B, TGFB1
**IL-8 Signaling**	9.97	0.0667	1.508	CDC42, EGFR, GNA13, GNAI2, GNAQ, GNAZ, RAC1, RAC2, RAP1A, RAP1B, RHOA, VCAM1
**NAD Signaling Pathway**	2.51	0.0331	1.342	ACADL, ACADM, LDHA, LDHB, TGFB1
**CLEAR Signaling Pathway**	1.41	0.0175	1.342	EGFR, RAB7A, RAP1A, RAP1B, TGFB1
**Apelin Adipocyte Signaling Pathway**	5.66	0.0769	1.342	GNAI2, GPX1, GPX3, GSTM1, RAC1, RAC2, SOD1

Ratio refers to the number of proteins from the dataset that map to the pathway listed divided by the total number of proteins that map to the canonical pathway from within the IPA knowledgebase.

**Table 6:** Top 10 canonical pathways and related proteins in hypertension compared with wild-type mice.

Canonical Pathway	Log (*p* Value)	Ratio	Z-Score	Proteins
**Role of Pattern Recognition Receptors in Recognition of Bacteria and Viruses**	2.39	0.0321	2	C1QA, C1QB, C1QC, MBL2, TGFB1
**White Adipose Tissue Browning** **Pathway**	1.85	0.029	2	CAMP, LDHA, LDHB, THRB
**Semaphorin Neuronal Repulsive** **Signaling Pathway**	1.72	0.0265	2	MYL6, MYL9, RAC1, RHOA
**RHOA Signaling**	4.68	0.0565	1.89	ARPC2, ARPC4, ARPC5, GNA13, MYL6, MYL9, RHOA
**Regulation of Actin-based Motility by Rho**	7.04	0.0776	1.667	ARPC2, ARPC4, ARPC5, CDC42, MYL6, MYL9, RAC1, RAC2, RHOA
**Phagosome Formation**	8.09	0.0304	1.606	ARPC2, ARPC4, ARPC5, CDC42, IGHE, IGHG3, IGHM, IGKC, LCAT, MYH10, MYH11, MYH14, MYH9, MYL6, MYL9, RAC1, RAC2, RAP1A, RAP1B, RHOA, TLN1
**IL-8 Signaling**	9.79	0.0667	1.508	CDC42, EGFR, GNA13, GNAI2, GNAQ, GNAZ, MMP2, MYL9, RAC1, RAC2, RAP1A, RAP1B, RHOA, VCAM1
**ILK Signaling**	8.99	0.065	1.508	CDC42, FLNA, ILK, MYH10, MYH11, MYH14, MYH9, MYL6, MYL9, PARVB, RAC1, RAC2, RHOA
**Signaling by Rho Family GTPases**	7.46	0.0485	1.414	ARPC2, ARPC4, ARPC5, CDC42, GNA13, GNAI2, GNAQ, GNAZ, MYL6, MYL9, RAC1, RAC2, RHOA
**Actin Cytoskeleton Signaling**	14.30	0.0776	1.213	ARPC2, ARPC4, ARPC5, CDC42, FLNA, GNA13, KNG1, MYH10, MYH11, MYH14, MYH9, MYL6, MYL9, RAC1, RAC2, RAP1A, RAP1B, RHOA, TLN1

Ratio refers to the number of proteins from the dataset that map to the pathway listed divided by the total number of proteins that map to the canonical pathway from within the IPA knowledgebase.

## Data Availability

The data presented in this study are available on request from the corresponding author.

## Supplementary Materials

The supporting information can be downloaded at: https://www.mdpi.com/article/10.3390/ijms24054930/s1.
