# Supplementary material for: The Proteome of Circulating Large Extracellular Vesicles in Diabetes and Hypertension
Source: Int J Mol Sci. 2023 Mar 3;24(5):4930. doi: 10.3390/ijms24054930 (PMC10003702; doi:10.3390/ijms24054930)
Supplement: Supplementary file 1 [file ijms-24-04930-s001.zip › ijms-2085380-supplementary.pdf]

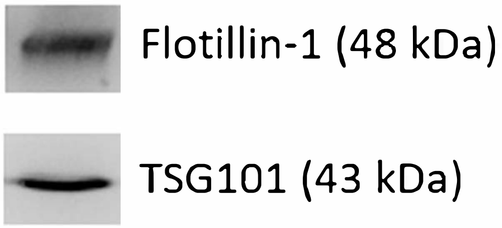

Supplemental Figure S1: Representative Western blot images of large EV isolates showing the presence of EV markers flotillin-2 and tumor suppressor gene-101 (TSG101).
